# Detection and Characterisation of Endosymbiont *Wolbachia* (Rickettsiales: Anaplasmataceae) in *Elaeidobius kamerunicus* (Coleoptera: Curculionoidea), Pollinating Agent of Oil Palm, and Its Relationships between Populations

**DOI:** 10.21315/tlsr2023.34.3.5

**Published:** 2023-09-30

**Authors:** Mohd Nur Azad Rushidi, Muhammad Luqman Hakim Azhari, Salmah Yaakop, Izfa Riza Hazmi

**Affiliations:** Centre for Insect Systematics, Department of Biological Science and Biotechnology, Faculty of Science and Technology, Universiti Kebangsaan Malaysia, 43600 Bangi, Selangor, Malaysia

**Keywords:** *Wolbachia*, *Elaeidobius kamerunicus*, endosymbiont, *pollinator*, oil palm, Malaysia, *Wolbachia*, *Elaeidobius kamerunicus*, Endosimbion, Pendebunga, Kelapa Sawit, Malaysia

## Abstract

*Elaeidobius kamerunicus* is the most efficient pollinator of oil palm. *Wolbachia* is an endosymbiotic bacteria associated with *E. kamerunicus* that has a potential to affect the fecundity and fitness of the *E. kamerunicus*. Despite their importance, no studies have been conducted to investigate its prevalence in *E. kamerunicus*. The objectives of this study were to detect and characterise *Wolbachia* in *E. kamerunicus* and determine the phylogenetic relationship of *Wolbachia* strains that infect *E. kamerunicus* by using three genetic markers namely Filamenting temperature-sensitive mutant Z (*ftsZ*), Chaperonin folding protein (*groEL*), and Citrate Synthase Coding Gene (*gltA*). DNA was extracted from 210 individuals of *E. kamerunicus* and the *Wolbachia* infections were detected using the *wsp* marker. The infected samples (*n* = 25, 11.9%) were then sequenced using *ftsZ, gltA* and *groEL* markers for strain characterization. In this study, a combination of four markers was used to construct the phylogeny of *Wolbachia*. Similar topologies were shown in all trees; Neighbour-Joining (NJ), Maximum Parsimony (MP), and Bayesian Inference (BI), which showed the mixing of individuals that harbor *Wolbachia* between populations. Interestingly, *Wolbachia* on *E. kamerunicus* was claded together with the species *Drosophila simulans* under supergroup B. This is the first report of *Wolbachia* infecting *E. kamerunicus* which is very valuable and significant as one of the parameters to evaluate the quality of the *E. kamerunicus* population for sustaining its function as a great pollinator for oil palm.

## Abstrak

*Elaeidobius kamerunicusis* merupakan pendebunga kelapa sawit yang paling berkesan. *Wolbachia* ialah bakteria endosimbiotik yang berhubung kait dengan *E. kamerunicus* dan berpotensi memberi kesan ke atas fekunditi dan kecergasan *E. kamerunicus*. Walaupun kepentingannya adalah besar, tiada kajian telah dijalankan untuk meneliti kelazimannya pada *E. kamerunicus*. Objektif kajian ini adalah untuk mengesan dan mencirikan *Wolbachia* pada *E. kamerunicus*, dan menentukan hubungan filogenetik strain *Wolbachia* yang menjangkiti *E. kamerunicus* dengan menggunakan tiga penanda genetik iaitu *Filamenting temperature-sensitive mutant Z* (*ftsZ*), *Chaperonin folding protein* (*groEL*) dan *Citrate Synthase Coding Gene* (*gltA*). DNA telah diekstrak daripada 210 individu *E. kamerunicus* dan jangkitan *Wolbachia* telah dikesan menggunakan penanda *wsp*. Sampel yang dijangkiti (*n*= 25, 11.9%) kemudiannya dijujuk menggunakan *ftsZ, gltA* dan penanda *groE*L untuk pencirian strain. Dalam kajian ini, gabungan empat penanda telah digunakan untuk membina filogeni *Wolbachia*. Topologi yang sama ditunjukkan dalam semua pohon; *Neighbour-Joining* (NJ), Parsimoni Maksimum (MP) dan Bayesian Inferensi (BI) yang menunjukkan percampuran individu *Wolbachia* antara populasi. Menariknya, *Wolbachia* pada *E. kamerunicus* terletak di klad yang sama dengan spesies *Drosophila simulans* di bawah superkumpulan B. Ini merupakan laporan pertama *Wolbachia* yang menjangkiti *E. kamerunicus* dan merupakan maklumat yang sangat penting sebagai parameter bagi menilai kualiti populasi *E. kamerunicus* dalam mengekalkan fungsinya sebagai pendebunga yang penting untuk kelapa sawit.

**Highlights**

- All sequences were confirmed using BLAST search tools, which reflected an identical percentage ranging from 98% to 100% for the *Wolbachia* species.
- The similar topologies in Neighbour-Joining (NJ), Maximum Parsimony (MP), and Bayesian Inference (BI) trees showed the mixing of individuals that harbour *Wolbachia* between populations.
- Based on the genetic distance analysis, the genetic distance values between *Wolbachia* in *E. kamerunicus* and *Drosophila simulans* (supergroup B) were approximately 0.02 and 0.03, respectively.

## INTRODUCTION

*Elaeidobius kamerunicus* Faust (Coleoptera: Curculionoidea) is identified as the most efficient insect pollinator of the oil palm (Yue *et al*. 2015). It was introduced in Cameroon, West Africa into the oil palm-growing countries in Southeast Asia in the early 1980s. It was also introduced to several countries parallel to the oil palm industry, including Malaysia, Thailand, Indonesia, Papua New Guinea and Colombia (Moslim & Kamarudin 2016).

A decade after introducing the *E. kamerunicus*, there were claims that fresh fruit bunch (FFB) yields had tremendously declined, causing several billion dollars in losses annually (Teo 2015). It might be due to poor pollination activity caused by inbreeding depression of the *E. kamerunicus* (Prasetyo *et al*. 2014); parasitism by a female nematode, *Elaeolenchus parthenonema* (Poinar *et al*. 2002); predators that modified their diets to prey on the *E. kamerunicus* (Muhammad Luqman *et al*. 2017); weather changes (Meléndez & Ponce 2016); and low quality of pollen carried by the *E. kamerunicus* (Teo 2015).

To date, no studies have investigated the prevalence of endosymbiotic bacterial infections in *E. kamerunicus* despite *Wolbachia*’s potential to affect the fecundity and fitness of the *E. kamerunicus*. *Wolbachia* might induce feminisation, parthenogenesis, male-killing or cytoplasmic incompatibility in its host. Therefore, it is believed that *Wolbachia* will give positive rather than negative effects, thus evolving to be mutualistic. In some cases, the host possibly has a low potential to survive after being disinfected. Some host species cannot reproduce, or even survive without *Wolbachia* colonisation. However, several studies have been conducted on other insect species, such as on *Wolbachia* in *Bactrocera* and other parasitoids species (Mohammed *et al*. 2017; 2018; Wan Mohd Nor *et al*. 2018).

The filamenting temperature-sensitive mutant Z (*ftsZ)* (Casiraghi *et al*. 2005), citrate synthase coding gene (*gltA)* (Dedeine *et al*. 2004), and heat shock protein (chaperonin group) (*groEL)* (Ros *et al*. 2009) gene markers were found effective in detection, screening, and obtain the strain-group, as well as for investigating their evolutionary relationships of *Wolbachia* strain (Bourtzis *et al*. 1996). Screening and detecting the *Wolbachia* in the *E. kamerunicus* species have become the priority to fill the research gap. It is because the information that will be generated is very useful and valuable as fundamental data for calculating the rate of *Wolbachia* infection in the *E. kamerunicus* species. It is essential to elucidate the potential effects of *Wolbachia* infection and explain the possible factors responsible for the decline or increase in the weevil population and its pollinating efficiency, which might have contributed to a lower or higher fruit set. Therefore, the objective of this study was to detect and characterise the presence of *Wolbachia* in *E. kamerunicus* and determine the phylogenetic relationship of *Wolbachia* strains that infect *E. kamerunicus* by using three genetic markers namely Filamenting temperature-sensitive mutant Z (*ftsZ*), Chaperonin folding protein (*groEL*), and Citrate Synthase Coding Gene (*gltA*).

## MATERIALS AND METHODS

### Samples Collection

A total of 210 individuals of *E. kamerunicus* were collected randomly (30 individuals per location) from seven different locations: Supa, Sarawak and Lahad Datu, Sabah representing the Borneo region (East Malaysia). Peninsular Malaysia has been represented by Keratong, Pahang (East Peninsular Malaysia); Banggol Rashid Sedim, Kedah (North Peninsular Malaysia); Kluang, Johor (South Peninsular Malaysia); Jengka, Pahang (Centre Peninsular Malaysia); and Teluk Intan (West Peninsular Malaysia). The random sampling has covered 1 hectare (100 m × 100 m), which a total of 30 trees (1 individual/spikelet/tree) selected for the molecular work. The variety of locations was aimed to elicit the differences in the presence of *Wolbachia* in the *E. kamerunicus* samples. The samples of *E. kamerunicus* were collected from male flowers when the oil palm was in the anthesis stage. The flowers were gently cut in the morning between 8.00 a.m. and 9.00 a.m. (which was not within the weevils’ active periods) for accessible collection. Insects were placed into plastic containers (24.5 cm × 13.5 cm × 13.0 cm). The spikelet of the male flowers was returned to the Cytogenetic laboratory in the Faculty of Science and Technology, Universiti Kebangsaan Malaysia (UKM). The adult samples of *E. kamerunicus* were preserved in 90% ethanol prior to DNA extraction.

### DNA Extraction

DNA extraction was performed using Nucleospin (Macherey-Nagel, German) extraction kit, following the standard operating procedure stated by the supplier. The whole body of *E. kamerunicus* was used in the extraction procedure to obtain a sufficient sample for the analysis. The extraction procedure required 40 μL MG buffer added with 10 μL of liquid proteinase K into the sample column and agitated for 20 min. Then, 600 μL of MG buffer was added and spun at 11,000 revolutions per minute (rpm). Before cleaning the lid and cell debris around the column wall, a high rotation speed was required. The solution was then transferred into a new collecting column using a filter collection tube to wash the silica membrane procedure. BW solutions were used for the first wash after the 11,000 rpm spin, followed by B5 solution for the second spin. In each step, all flow through in the collecting tube was discarded. Finally, 100 μL of elution buffer was added to the collection tube to obtain a highly pure DNA sample.

### PCR Amplification

The *wsp* gene marker was used to detect the presence of *Wolbachia*. The sequences used to amplify DNA fragments were 81F 5′ TGG TCC AAT AAG TGA TGA AGA AAC-3′ and 691R 5′-AAA AAT TAA ACG CTA CTC CA-3′. The DNA fragment′s length produced was approximately 600 bp (Zhou *et al*. 1998). The *ftsz, gltA* and *groEL* markers were characterised and used for constructing the phylogenetic trees of *Wolbachia*-infected *E. kamerunicus* with other infected species. All the target regions were amplified using *ftsZ* (Dedeine *et al*. 2004), forward 5′-TTG CAG AGC TTG GAC TTG AA-3′, reverse 5′-CAT ATC TCC GCC ACC AGT AA-3′; *gltA* (Casiraghi *et al*. 2005), WgltAF 5′-TAC GAT CCA GGG TTT GTT TCT AC-3′, WgltAR 5′-CTC ATT AGC TCC ACC GTG TG-3′; and *groEL* (Ros *et al*. 2009), forward 5′-CAA CRG TRG SRR YAA CTG CDG G-3′, 5′-GAT ADC CRC GRT CAA AYT GC-3′.

PCR amplification was conducted in 25 μL for the final volumes consisting of 12.5 μL Go*Taq* Green master mix PROMEGA, 7.2 μL ddH20, 1.0 μL forward primer, 1.0 μL reverse primer, and 3.0 μL DNA sample. The thermal condition was constant for each primer: pre-denaturation at 95°C/3 min, denaturation at 95°C/30 sec, pre-elongation at 72°C/30 sec for 30 cycles, and the final elongation at 72°C/10 min. The annealing temperatures for *wsp*, *fts, gltA* and *groEL* were 53.0°C, 55.0°C, 53.0°C and 49.0°C respectively (Mohammed *et al*. 2017).

### Sequencing Analysis

All the PCR techniques were detected by amplifying the expected results. The PCR products were run in 1.5% agarose gel and observed under ultraviolet light. Only samples with a positive result from *wsp* were amplified with *ftsZ, gltA* and *groEL* for sequencing. The PCR products were purified and sequenced by Apical Scientific Sdn. Bhd. The sequencing results obtained were checked using the Basic Local Alignment Tool (BLAST) for species confirmation.

### Sequences Alignment and Phylogenetic Analyses

#### Supergroup determination

Based on the data of the *groeL*, the phylogeny using NJ based on the Kimura-2 parameter was constructed to determine the supergroup of EK *Wolbachia*. Representatives of *Wolbachia* from three localities that were detected harbouring *Wolbachia* (T1 3B, KT 3B and JK 2B) were used along with other four individuals from different groups of insects obtained from the Genbank: termite (*Kalotermes flavicollis*), pteromalid wasp (*Nasonia giraulti*), fruit fly (*Drosophila simulans*), and flea (*Ctenocephalides felis*) were used to constructing the phylogeny tree and determine the supergroup of *Wolbachia* in EK. Genetic distance between species was measured using MEGA 3.1 (Kumar *et al*. 2004). In addition, only three samples for each locality were included because based on earlier genetic analysis (all individual samples), too small variation or almost none different was detected. Therefore, only three representatives each locality included for supergroup clade and genetic distance.

#### Phylogenetic analyses based on combination of genes

The sequencing results were edited and aligned using Sequencer and MEGA7 software. Neighbour-Joining (NJ), Maximum Parsimony (MP), and Bayesian Inference (BI) were implemented to construct the phylogenetic trees. All sequences were combined and analysed using Incongruence Length Differences (ILD) in PAUP 4.0b (Swofford 2002). The NJ analysis was run using the Kimura-2 parameter (2KP) with 1000 bootstrap replications. A parsimony tree was constructed using a heuristic search with Tree Bisection Reconnection (TBR), 1,000 stepwise addition replicates to obtain a 50% majority-rule consensus tree constructed with bootstrap 1000 replications (Swofford 2002). PAUP 4.0b10 was employed to run the NJ and MP trees. Meanwhile, Bayesian Inference (BI) trees were constructed using MrBayes 3.1.2 software (Ronquist *et al*. 2012). The best-fit model was selected using jModeltest 3.7 based on Akaike Information Criterion (AIC). BI tree was generated using two chains of Monte Carlo Markov Chain (MCMC).

## RESULTS

### *Wolbachia* Detection

A total of 210 individuals of *E. kamerunicus* were screened using *wsp*. Only 25 samples were detected with *Wolbachia* infection, represented by 10 males and 15 females (12%) (Table 1). All sequences were confirmed using BLAST search tools, which reflected an identical percentage ranging from 98% to 100% for the *Wolbachia* species. Based on the results from the three genes, 21 successful individuals were amplified using the *ftsZ* marker, 20 individuals using the *gltA* marker, and 19 individuals using the *groEL* marker. The summary of the total DNA that was successfully amplified by using all three markers is shown in Table 2.

### Classification of *Wolbachia* Supergroup

**Figure 1:**
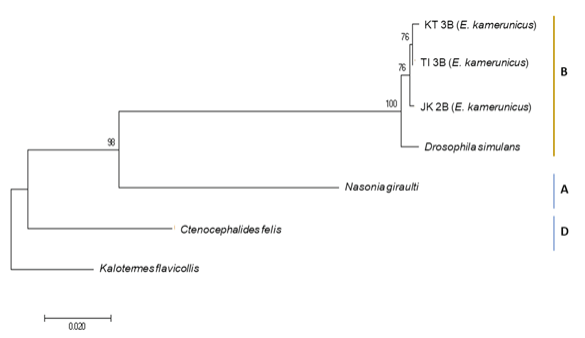
The Neighbour-Joining tree between *Wolbachia* individuals in *E. kamerunicus* and other species by using the *groEL* marker

The *groEL* marker was used to support information of the *wsp* gene marker that the *Wolbachia* strain infected *E. kamerunicus* was from a supergroup B. This was executed by constructing the phylogeny tree using the *groEL* gene information. *Kalotermes flavicollis* was selected as the outgroup for the phylogeny tree (Fig. 1). The *Wolbachia* clade inside *E. kamerunicus* was observed to be together with *Wolbachia* species inside *D. simulans* with a bootstrap support value of 100%. *Nasonia giraulti* and *Ctenocephalides felix* were separated with a support value of 100%.

Based on the genetic distance analysis, the genetic distance values between *Wolbachia* in *E. kamerunicus* and *D. simulans* (supergroup B) were approximately 0.02 and 0.03, respectively. In comparison, the genetic distance between *E. kamerunicus* and *Nasonia giraulti* (supergroup A) was 0.9, while that of *Ctenocephalides felix* (supergroup D) was 1.0. Therefore, the small genetic distance between *E. kamerunicus* and *D. simulans* (supergroup B) supports the idea that the *Wolbachia* strain in this study was grouped with supergroup B (Table 3).

### Tree Reconstruction

**Figure 2:**
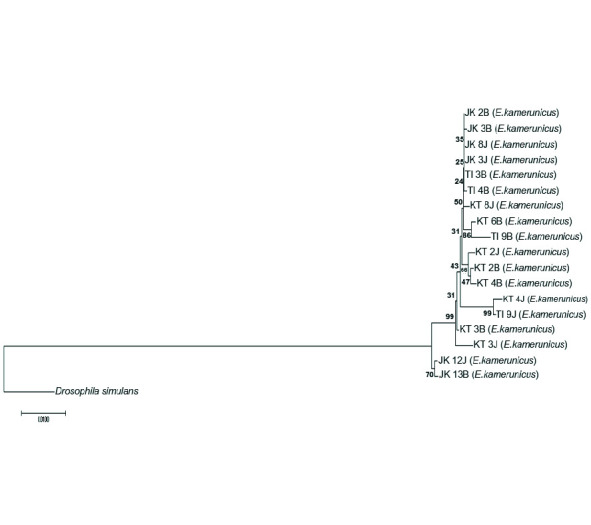
The NJ phylogeny tree by combining sequences from the *ftsZ*, *gltA* and *groEL*.

**Figure 3:**
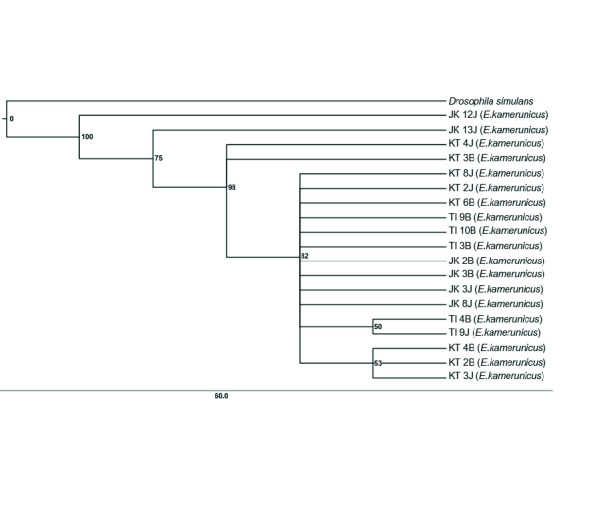
The MP phylogeny tree by combining the sequences from *ftsZ*, *gltA* and *groEL*

**Figure 4:**
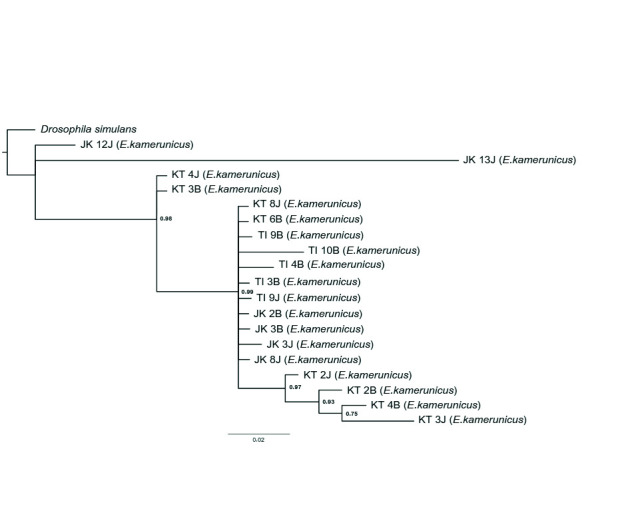
The BI phylogeny tree by combining sequences from *ftsZ*, *gltA* and *groEL*

The significant value from the ILD test shows that values below 0.05 (< 0.05) were suitable and approved for the combination of marker genes. The distance criteria had been set to construct the NJ tree on the combination tree The NJ tree depicts that one clade has formed even though an individual *E. kamerunicus* from Jengka, Pahang, was separated from the clade (Fig. 2). Small groups were also formed inside the clade, such as individuals from Keratong, Pahang and Teluk Intan, Perak. The support values of the group were 51% and 57%, respectively. Some individuals separated the branches from Jengka, Pahang from the big clade (Fig. 3). The bootstrap value of individual relations inside the clade and individuals from Jengka, Pahang that was isolated was 100%. The supporting bootstrap values on the respective inner branches were between 50% and 98%. A single clade was formed for the Bayesian inference tree, but several *E. kamerunicus* individuals were grouped from Jengka, Pahang. The posterior probabilities (PP) value of individual parts was supported between 0.75 p.p and 0.98 p.p (Fig. 4).

The topology of a phylogenetic tree that combines sequences of regions *ftsZ, gltA* and *groEL* has shown the form of soft polytomy trees. All the tree topologies are similar, which separated two individuals, male EK (Jengka, Pahang) and female EK (Jengka, Pahang), from the rest of the infected samples in MP and BI trees (Figs. 3 and 4). However, for the NJ tree (Fig. 1), only the male EK (Jengka, Pahang) separated and emerged earlier compared to the rest. There was no separation (mixture) between all the *Wolbachia* strains between localities.

Based on the topology of the phylogenetic tree constructed using three construction methods, the combined phylogeny tree was observed to provide important information and a good tree divergence. However, this phylogeny information is imperfect and needs to be improved using more gene regions and combined data to further understand the relationship between individuals and different species infected by *Wolbachia*. In addition, the information on *Wolbachia* evolution that infects a particular host is also required to understand their adaptation process inside the host’s body and study their relations and survival.

## DISCUSSION

This study employed the *wsp* marker in detecting the infection process that has reported the reliability of this gene marker by showing 100% on the infected or PCR-positive samples (*n* = 25). The effectiveness of the *wsp* to make detection on *Wolbachia* in several hosts was supported by the study of *Bactrocera* spp. (Mohammed *et al*. 2017) and *Drosophila auraria* (Diptera: Drosophilidae) by Parvizi *et al*. (2013). Additionally, the *wsp* marker is also compatible with investigating the relationship of evolution in multiple strains of *Wolbachia* in *Drosophila* species that identifies the effects of cytoplasmic incompatibility on the progenies (Bourtzis *et al*. 1996).

All three markers (*ftsZ*, *gltA* and *groEL* genes) had shown their abilities to prove the divergence between the *Wolbachia* strains infecting a wide variety of species by constructing a phylogeny tree (Fenollar *et al*. 2003; Schulenburg *et al*. 2000). Combining genes is fundamental to get a more precise and accurate result. Although only 25 samples were successfully detected with *Wolbachia* using the *wsp* marker, a combination of *ftsZ, gltA* and *groEL* presented more informative findings in this study, especially for the phylogenetic study. According to some studies, using more markers to detect *Wolbachia* could lead to more inconsistent and inaccurate results. Nine different types of genes markers (16S *rRNA, gltA, groEL, wsp, gatB, fbpA, coxA, hcpA* and *ftsZ*) were used to study the *Wolbachia* in galling aphid species (Hemiptera: Aphidoidea) by Ren *et al*. (2020), but the detection results varied for each individual.

Based on the sex ratio, this study found that female *E. kamerunicus* weevil revealed a higher infection rate of 60% compared to 40% in male. It is probably due to the maternal inheritance that has caused the manipulation of the sex ratio to become significant and occur naturally (Kageyama *et al*. 2002). Other than that, the female-biased condition creates the mechanism of male-killing or feminisation in *Wolbachia*. This is one of the causes of gender ratio disruption that occur in an infected species population, which is similar to the events reported among *Tribolium confusum* (flour beetle) (Coleoptera: Tenebrionidae) (Fialho & Stevens 2000) and *Ostrinia scapulalis* (Adzuki seed borer) (Lepidoptera: Crambidae) (Sugimoto & Ishikawa 2012).

Based on the phylogeny, it could be concluded that the *Wolbachia* in the *E. kamerunicus* is specifically from supergroup B, which resembled that of the *Wolbachia* found in the model moth species, *Eurema hecabe*. They are probably similar and transform genetic males into females, consequently increasing the number of individuals in the population (Narita *et al*. 2007). In this study, the *groEL* gene region has provided a higher informative value than other regions. The *groEL* region is able to show better genetic diversity compared to the 16S rRNA gene and provides better advantages in the construction of the phylogeny tree for the bacterial endosymbiont species such as *Orienta tsutsugamushi* (Rickettsiales: Rickettsiaceae) (Arai *et al*. 2013). In fact, the *groEL* region is able to show the molecular differences of *Bacillus cereus* and constructs a better phylogeny tree with a higher bootstrap support value (Chang *et al*. 2003). The successful classification of strain groups in *Wolbachia* A–D has also been shown by Casiraghi *et al*. (2005) using the *groEL* gene region.

This study detected *Wolbachia* infection in three out of seven oil palm plantations in various states. The displacement environment of *E. kamerunicus* weevil could occur either through migration or sharing the weevil’s resources for agriculture. On the other hand, the mechanism of endosymbiont bacteria might infect the hosts through the mechanism of inheritance (vertical) and species that are present in other populations. Population genetic and demographic analyses were vital in this study to understand the diversity of this strain according to different locations. Both analyses confirmed the relationship between *Tamarixia radiata* parasitoid (Hymenoptera: Eulophidae) and *Wolbachia* in the population and between different regions (Ashraf *et al*. 2021).

Nevertheless, the infection rate in the population of *E. kamerunicus* should be a concern given the significance of the oil palm pollinator beetle to the oil palm ecosystem and production. The effect of cytoplasmic incompatibility and feminisation will threaten and harm oil palm productivity in the country. A study of *Wolbachia’s* transmission rate to *D. simulans* showed 100 km per year and successfully changed the rare status to fully infected in only three years (Turelli & Hoffman 1991). Likewise, Weeks *et al*. (2007) also disclosed a faster transmission rate in population *D. simulans* with a distance of 700 km in several years, though the reproduction system of females had been manipulated.

Hard or soft polytomy trees could be formed when information is not sufficient to build characterisation analysis and if there is too many branching on one ancestry node (hard polytomy) (Lin *et al*. 2011). Unfortunately, in the present study, the information from the evolution value on the phylogeny tree is shallow and does not give a better view of the divergence of infected species. However, in a study that uses microbe samples, the condition of polytomy can be said to be possible, as well as for this study. This is due to microbe characteristics, which are not varied in terms of morphological features, and having the characteristics of a shallow rate of evolution that it poses difficulty to differentiate the phylogeny tree by multifurcations or bifurcations.

In this study, a combination of trees has successfully shown a better informative region than a single tree and formed a more diverging tree (Figs. 3, 4 and 5). The similarities or merging of information between the *Wolbachia* strain between different populations in this study can still be observed in the outcome of combined data. Combining various gene regions can help obtaining broader information about gene diversity and the genetic population (Kovanen *et al*. 2014). Besides, a precise phylogeny tree helps to observe new species’ existence, detect a new adaptation at the molecular level, understand morphological characteristics’ evolution, and build demographic changes on new diverging species (Kapli *et al*. 2020). More information could increase the bootstrap value and information about the phylogeny tree (Susko 2016).

Though the isolation of the population was expected to form according to the respective population in the early stage or hypothesis, several things may happen due to higher gene flow between *Wolbachia* populations within *E. kamerunicus*. This matter might be due to the behavior of this flying species between locations or the plantations that were said to be near and inside a small spatial distance (scale) (Keratong and Jengka, Pahang). According to specific agriculture companies, the source of oil palm samplings could also contribute to the ability of gene displacement happening between plantations of different regions.

The *E. kamerunicus’* ability to fly, high activity in the rainy season, and breed are better than other *Elaeidobius* spp. (i.e., *E. plagiatus, E. subvittatus*, and *E. singularis*), thus allowing *Wolbachia* to spread at a higher rate (Daud & Ghani 2016; Siswanto & Soetopo 2020; Yue *et al*. 2015). Several similar conditions occur in bagworms, *Metisa plana*, where individuals from various populations mix despite the remarkable difference in the distance between regions (Badrulisham *et al*. 2021). In addition, some studies showed apparent isolation between populations of the same species at a considerable distance involving regions with different weather conditions and geographic factors that could facilitate different parasitism rates (Ashraf *et al*. 2021). The movement of *Wolbachia* across other species has been identified through vertical transmissions to most of the arthropod species by maternal inheritance (Duplouy *et al*. 2015). Meanwhile, horizontal transmission has been stated to rarely occur. However, the mechanism of transmission could be elicited either through the displacement of bacteria in the laying area (Huigens *et al*. 2004) or through a plant as a medium (shelter or food) (Li *et al*. 2017). In the present study condition for *E. kamerunicus*, transmission between individuals would likely occur through both displacement methods. Transmission through maternal inheritance has been proven by Kageyama *et al*. (2012) on Lepidoptera species, whereas transmission through habitat shelter was identified on stinger Braconidae species (Schuler *et al*. 2016) and *Bemisia tabaci* (whitefly) (Li *et al*. 2017).

## CONCLUSION

The ability of *Wolbachia* to infect oil palm-pollinating weevils has generated interest in its usage to cause femininity and fecundity in *E. kamerunicus*. More research is needed to improve the body of knowledge on the benefits of the symbiosis between *Wolbachia* and *E. kamerunicus*. In order to detect a wider range of *Wolbachia* infections, studies need to use larger sample sizes and select more plantation areas. Another common technique for *Wolbachia* study such as Multi Locus Sequence Technique (MLST) should be conducted in order to obtain more extensive information on Wolbachia strains, the relationship between strains, and the effects of infection. Through MLST, information related to the studied species could produce more specific results such as the diversity of strains, group isolation according to molecule information, and the relationship between strains studied. Such vital information will assist in enhancing sustainable oil palm production, thus boosting the production of one of the leading natural commodities of Malaysia.

## Figures and Tables

**Table 1:** Total infected *E. kamerunicus* in seven localities from Malaysia.

Locality	Peninsular Malaysia					Borneo		

	North	West	East	Central	South	Sabah	Sarawak	Total
Tag	Sedim, Kedah	Teluk Intan, Perak	Keratong, Pahang	Jengka, Pahang	Kulai, Johor	Lahad Datu	Lupa, Sarawak	
+Male	–	3	4	3	–	–	–	10
+Female	–	7	5	3	–	–	–	15
+Total	–	10	9	6	–	–	–	25

**Table 2:** The individuals infected with *Wolbachia*, the types of gene marker used, and species that were successfully amplified.

Location	Specimen code	Genes			

		*wsp*	*ftsZ*	*gltA*	*groEL*
East Peninsular Malaysia: Keratong, Pahang	KT 2J	+	/	/	/
	KT 3J	+	/	/	/
	KT 4J	+	/	/	/
	KT 8J	+	/	/	/
	KT 2B	+	/	/	/
	KT 3B	+	/	/	/
	KT 4B	+	/	/	/
	KT 6B	+	/	/	/
	KT 12B	+	/	−	−
West Peninsular Malaysia: Teluk Intan, Perak	TI 2J	+	−	−	−
	TI 9J	+	/	/	/
	TI 10J	+	−	−	−
	TI 2B	+	/	/	−
	TI 3B	+	/	/	/
	TI 4B	+	/	/	/
	TI 5B	+	−	−	−
	TI 7B	+	−	−	−
	TI 9B	+	/	/	/
	TI 10B	+	/	/	/
Centre Peninsular Malaysia: Jengka, Pahang	JK 3J	+	/	/	/
	JK 8J	+	/	/	/
	JK 12J	+	/	/	/
	JK 2B	+	/	/	/
	JK 3B	+	/	/	/
	JK 13B	+	/	/	/

Total		25	21/25	20/25	19/25

*Notes*: + = infected with *Wolbachia;* / = sequenced; − = not sequenced

**Table 3:** Genetic distance using the *ftsZ* marker for the *Wolbachia* in *E. kamerunicus* and other species.

		1	2	3	4	5	6
1	*K. flavicollis*	–					
2	*N. giraulti*	0.10345	–				
3	*D. simulans*	0.11434	0.07985	–			
4	*C. felix*	0.08955	0.09886	0.10862	–		
5	*TI 3B*	0.12699	0.09099	0.02354	0.10958	–	
6	*KT 3B*	0.13378	0.09814	0.03136	0.11648	0.00000	–
7	*JK 2B*	0.12699	0.09099	0.02354	0.10958	0.00000	0.00000
